# Combinations of Blue and Red LEDs Increase the Morphophysiological Performance and Furanocoumarin Production of *Brosimum gaudichaudii* Trécul *in vitro*

**DOI:** 10.3389/fpls.2021.680545

**Published:** 2021-07-21

**Authors:** Érica Letícia Gomes Costa, Fernanda dos Santos Farnese, Thales Caetano de Oliveira, Márcio Rosa, Arthur Almeida Rodrigues, Erika Crispim Resende, Ana Helena Januario, Fabiano Guimarães Silva

**Affiliations:** ^1^Departamento de Biotecnologia, Instituto Federal de Educação, Ciência e Tecnologia Goiano, Rio Verde, Brazil; ^2^Laboratório de Fisiologia Vegetal, Instituto Federal de Educação, Ciência e Tecnologia Goiano, Rio Verde, Brazil; ^3^Faculdade de Agronomia da Universidade de Rio Verde, Rio Verde, Brazil; ^4^Departamento de Biomoléculas, Instituto Federal de Educação, Ciência e Tecnologia Goiano, Iporá, Brazil; ^5^Núcleo de Pesquisa em Ciências Exatas e Tencológicas, Universidade de Franca, São Paulo, Brazil

**Keywords:** spectral quality, photosynthetic apparatus, phenolic compounds, psoralen, bergapten

## Abstract

*Brosimum gaudichaudii* is a plant species with medicinal relevance due to its furanocoumarin accumulation. The accumulation of these compounds in the root promotes predatory extractivism, which threatens the conservation of the species. In addition, little is known about the conditions for culturing of this species *in vitro*. The present study aimed to investigate how the application of different spectra of LEDs (white, blue, red, and combinations of blue and red at 1:1 and 3:1 ratios) can impact the morphophysiological and biochemical characteristics of *B. gaudichaudii* under different *in vitro* conditions. To evaluate the production of furanocoumarins in its leaves, which are easy-to-collect perennial organs, we cultured nodal segments in 50-mL tubes with MS medium under 100 μmol m^−2^ s^−1^ light and a photoperiod of 16 h for 50 days. We then submitted the seedlings biometric, anatomical, biochemical, and physiological evaluations. The different spectral qualities influenced several characteristics of the seedlings. Plants grown under red light showed greater stem elongation and larger and thinner leaves, strategies aimed at capturing a higher ratio of radiant energy. Exposure to the blue/red ratio of 1:1 induced increases in the concentration of the furanocoumarin psoralen, probably due to the diversion of carbon from primary metabolism, which resulted in lower growth. Cultivation under blue light or blue:red light at 3:1 triggered anatomical and physiological changes that led to higher production of secondary metabolites in the leaves, and at the 3:1 ratio, the seedlings also had a high growth rate. These results highlight the fundamental role of light in stimulating the production of secondary metabolites, which has important implications for the production of compounds of interest and indirect consequences for the conservation of *B. gaudichaudii*.

## Introduction

In Brazil, scientific approaches to the cultivation of medicinal Cerrado species are still limited, even though it is an extremely diverse and heterogeneous biome. In fact, only 8% of the 55.000 cataloged plant species have been studied for the presence of bioactive compounds, and only 1.100 of the species have been evaluated for their medicinal properties (Nunes et al., 2018; Ribeiro Neto et al., 2020). Among the native Brazilian species with medicinal potential is *Brosimum gaudichaudii* Trécul, a tree species that can reach up to 10 m in height. It belongs to the Moraceae family and is popularly known as mama-cadela. Recent studies have identified this species as one of the most promising in the treatment of autoimmune skin diseases due to its high production of furanocoumarins, and there is already a patent application for the development of drugs using *B. gaudichaudii* (Ribeiro et al., 2017; Morais et al., 2018; Quintão et al., 2019). Of all compounds involved in the treatment of depigmentation disorders, linear furanocoumarins (psoralen and bergapten) are the most used in clinical trials, as they have been combined with ultraviolet radiation in the treatment of diseases such as psoriasis, vitiligo, eczema, alopecia, primary T-cell lymphomas (fungal mycosis), and central nervous system and circulatory system disorders (Sumorek-Wiadro et al., 2020; Zhao et al., 2020). Psoralen and bergapten, as well as other furanocoumarin compounds, exert their photosensitizing effects by covalently bonding DNA, which is triggered by light of specific wavelengths (320–410 nm) (da Silva et al., 2009).

Psoralen and bergapten can be obtained from plant extraction or chemical synthesis, but the former is preferred because the synthesis of furanocoumarins is expensive and generates toxic waste (Martins et al., 2015a). Plant extraction, however, threatens the conservation of the species. In fact, the medicinal extracts prepared from *B. gaudichaudii* mainly use the root of this plant, the organ that accumulates the highest concentrations of linear furanocoumarins (mainly psoralen and bergapten) (Ribeiro et al., 2017; Quintão et al., 2019; Sumorek-Wiadro et al., 2020), while other organs, such as the stem and leaves, have trace amounts of these compounds, with concentrations up to 10 times lower than those observed in the roots (Melo, 2004). The allocation of so much of the furanocoumarins in the roots limits the use of other parts of the plant and makes extractivism predominantly predatory since it is necessary to remove the root system to obtain the medicinal compound (Silva et al., 2011; Coradin et al., 2016). It should also be noted that although there are some commercial plantations of *B. gaudichaudii*, the traditional cultivation does not meet the growing market demand due to its low biomass, long growth cycle, and high consumption (Martins et al., 2015a). It is easy to see that the future supply of furanocoumarins will depend on the development of biotechnologies that improve the cultivation of species that produce and accumulate psoralen and bergapten, as well as methods that increase the production of these compounds in different plant organs. Indeed, if it is possible to increase the production of psoralen and bergaptene in the leaves, perennial organs of rapid production, a single plant could be used repeatedly to collect the compounds, favoring the sustainable use of furanocoumarins and contributing to the conservation of the species.

*In vitro* culturing is one of the methods with the greatest potential for *ex situ* conservation of plant species with medicinal properties, as it allows rapid multiplication of plants and large-scale production (Fallah et al., 2019). In addition, *in vitro* culture conditions can be controlled to maximize the production of bioactive compounds of interest (Veraplakorn, 2016). Light is a key factor in this process since it regulates several physiological processes, directly or indirectly influencing the primary and secondary metabolism of plants. Thus, variations in light conditions may result in different growth responses and metabolite production (Alvarenga et al., 2015). In fact, irradiance with photons of different wavelengths is an essential abiotic component required by plants for photosynthesis, growth, and accumulation of secondary metabolism products, with blue and red light being particularly important in these processes (Li et al., 2020).

One of the most effective ways to analyze the influence of light on the morphological, anatomical, and metabolic characteristics of plants is to use light-emitting diodes (LEDs; Silvestri et al., 2019; Chen et al., 2020). LEDs have numerous advantages over traditional lighting methods, such as low cost, small size, high energy efficiency, low heat emission, and strict control of the spectral composition, which allows the culture of plants at specific wavelengths (Monostori et al., 2018; Silva et al., 2020). The culture of plants using blue and red light plays an important role in the development and growth of the photosynthetic apparatus by regulating biomass production and allocation, plant growth, and antioxidant system activation. The combination of blue and red light promotes an increase in these responses when compared to monochromatic treatments (Dutta Gupta and Karmakar, 2017; Zhang et al., 2018). The use of these light qualities also influences the production of several classes of secondary metabolites, such as linear furanocoumarins, by acting on the expression of genes of the biosynthetic pathway, which are highly inducible and efficiently stimulated by blue light (Kitamura et al., 2005; Szopa et al., 2012; Kubica et al., 2020).

Based on the above and considering the risk that predatory extractivism poses to the conservation of *B. gaudichaudii*, the present study aimed to investigate the changes triggered by light in the physiology and anatomy of this species, verifying how these changes favor the production of furanocoumarins in *B. gaudichaudii* seedlings cultivated *in vitro*. Although the roots are the organ traditionally associated with furanocoumarin accumulation, this study focused on leaves, which are easy-to-collect perennial organs, aiming to provide a viable and sustainable alternative for the commercial use of this species. The following hypotheses were tested: (i) the combination of different wavelengths favors the growth and development of *B. gaudichaudii* seedlings, contributing to more efficient seedling production; and (ii) the combination of different wavelengths activates secondary metabolic pathways, increasing the production of furanocoumarins in the leaves, enabling this organ to become a source of medicinal compounds. The results obtained are unprecedented and demonstrate the great ability of *B. gaudichaudii* seedlings to adapt to variations in the spectra provided by LED lamps *in vitro* by increasing their production of furanocoumarins in the leaves, which has important implications for obtaining these compounds in a more sustainable way.

## Materials and Methods

### Plant Material and Experimental Conditions

Ripe mama-cadela fruits were collected between September and November 2018 from adult plants in the municipality of Montes Claros de Goiás (16°10′8″ S, 51°27′12″ W, 412 m altitude), Brazil. After collection, the fruits were pulped to obtain the seeds. Next, the seeds were sown in plastic trays containing the commercial substrate Bioplant^®^. The trays were kept in a growth room at 25 ± 3°C for 45 days to obtain the explants.

### *In vitro* Establishment of *Brosimum gaudichaudii* Seedlings

At 45 days of sowing, healthy and homogeneous *B. gaudichaudii* seedlings were selected, and nodal segments measuring approximately 2.0 cm and containing two shoots were used for *in vitro* establishment. The segments were kept in water with three drops of detergent (Tween 20) for 20 min. Then they were immersed in 70% ethyl alcohol for 1 min in a laminar flow hood, followed by commercial 50% sodium hypochlorite (NaOCl) for 30 min, and then washed three times in autoclaved water.

After disinfection, the nodal segments were cultured in test tubes (25 × 150 mm) containing 20 mL of Murashige and Skoog (MS) medium (Murashige and Skoog, 1962), supplemented with 30 g L^−1^ sucrose, 3.5 g L^−1^ agar, 2 g L^−1^ activated charcoal and 30 μM 6-benzylaminopurine. The pH was adjusted to 5.7 ± 0.03 before the addition of the gelling agent. The culture medium was autoclaved at 120°C for 20 min and then used for inoculation. The tubes containing the nodal segments were kept in a growth room under a photoperiod of 16 h at 50 μmol m^−2^ s^−1^ and temperature of 25 ± 3°C for 30 days.

### Growth Conditions

After 30 days, the explants were selected and subcultured in test tubes containing 20 mL of MS medium supplemented with 30 g L^−1^ sucrose, 3.5 g L^−1^ agar, 2 g L^−1^ activated charcoal, and 30 μM 6-benzylaminopurine.

**Figure 1:**
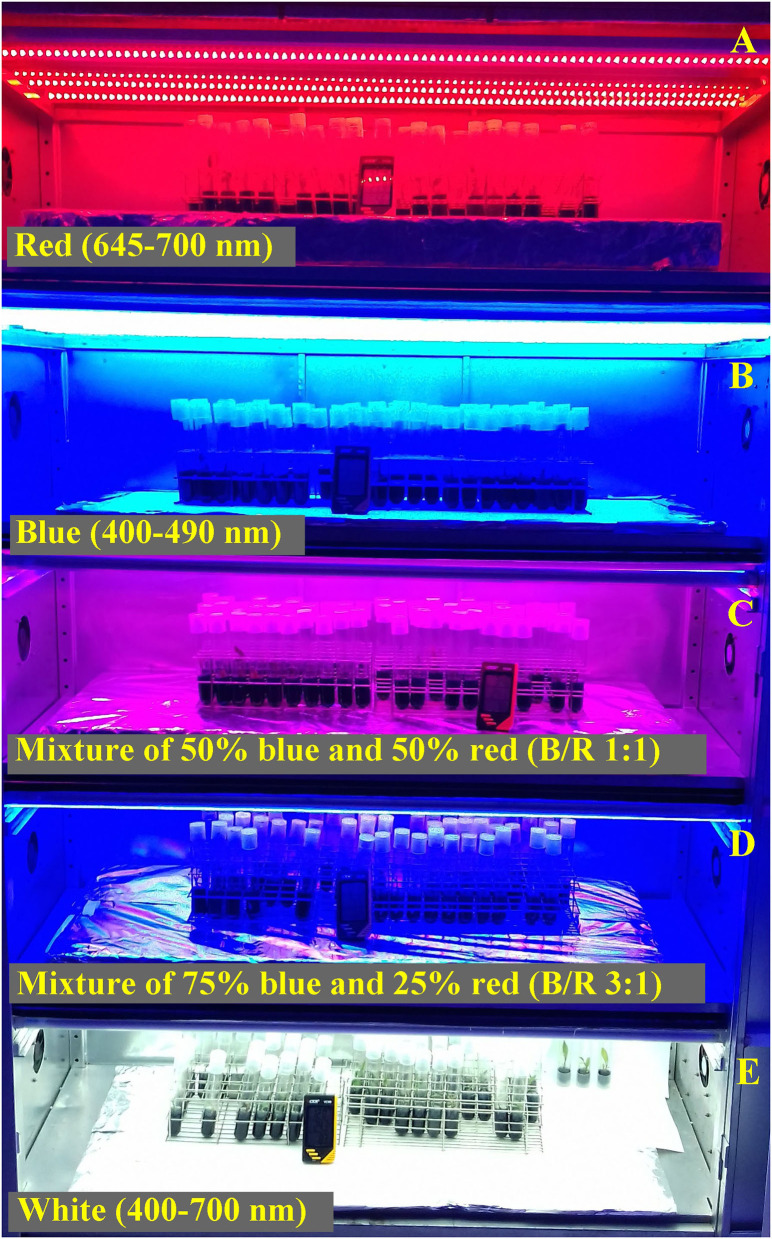
Relative spectral distribution of LEDs: red (645–700 nm) **(A)**; blue (400–490 nm) **(B)**; mixture of 50% blue and 50% red (B/R 1:1) **(C)**; mixture of 75% blue and 25% red (B/R 3:1) **(D)**, and white (400–700 nm) **(E)** used in the *in vitro* culture of *Brosimum gaudichaudii*.

The experiment was carried out in a completely randomized design, with five treatments (different light conditions) and 66 explants per treatment. The treatments consisted of 20 W LED lamps (Lanao serie Tubes, China) with different monochrome or combined wavelengths: (i) white (400–700 nm), (ii) blue (400–490 nm), (iii) red (645–700 nm), (iv) mixture of 50% blue and 50% red (B/R 1:1) and (v) a mixture of 75% blue and 25% red (B/R 3:1), respectively (Figure 1). The light spectra and the characteristics of the ratio between blue and red were calculated by defining the relative areas of the spectrum within the regions using a portable USB2000 spectroradiometer (Ocean Optics, Dunedin, FL, USA), and the collected data were processed by using the PARSpec Module application coupled with the SpectraSuite software (spectra shown in Supplementary Figure 1). The photosynthetically active radiation was determined with a PAR sensor, model APG-SQ-316 (Apogee, North Logan, UT, USA) (Pennisi et al., 2019). The photosynthetic photon flux density was fixed at 100 ± 5 μmol m^−2^ s^−1^, photoperiod of 16 h, temperature of 25 ± 3°C, and 40 ± 10% relative humidity.

After a 50 days growing period under the respective light sources, 30 seedlings per treatment were selected according to uniformity of size and morphology. The experiment was conducted only once and each treatment (light conditions) had between five and ten repetitions, according to the specificity of each analysis. Five different repetitions were analyzed for practically all parameters and, due to the availability of biomass, only chlorophyll, flavonoid starch, nitrogen balance index and photosynthetic analysis were performed using the same 5 seedlings. For the phytochemical analysis, samples composed of 10 replicates per treatment were used, in order to reach the necessary leaf mass.

### Analysis of Growth Parameters

To characterize the effect of light on plant growth parameters, shoot length (cm), number of leaves, leaf dry matter (mg), stem dry matter (mg), total dry matter (mg), leaf area (cm^2^), and specific leaf area (SLA) were measured. The leaf area was analyzed in ImageJ^®^ (Rasband, WS; ImageJ, Bethesda, MD, USA). To determine the biomass variables, the seedlings were dried at 70°C until reaching constant weight. The SLA was calculated as the leaf area in cm^2^/leaf dry matter (g).

### Estimation of Chlorophyll, Flavonol, and Anthocyanin Content and the Nitrogen Balance Index

Estimation chlorophyll, flavonoid (flavonols and anthocyanins) contents, and nitrogen balance index (NBI) were measured by means of a Dualex^®^ Scientific Polyphenols and Chlorophyll Meter (FORCE-A, Orsay, France). The instrument measures the chlorophyll amount by exciting the leaves with two radiations at different wavelength (red and near infrared) (Cerovic et al., 2012; Silvestri et al., 2019). At the same time, it calculates the flavonoids amount as a logarithmic ratio between infrared fluorescence of the chlorophyll excited by a red wavelength and by a UV wavelength. Indeed, flavonoids, located in epidermal cells, absorb UV light lowering the chlorophyll fluorescence in the infrared range. NBI is given as a ratio between the amounts of chlorophyll and flavonoids (Coelho et al., 2012). For this evaluation, readings were performed at the middle third of the adaxial portion of the leaves, the total chlorophyll, anthocyanins, and flavonoids content estimate.

### Analysis of the Chlorophyll *a* Fluorescence Kinetics

In the same leaves used to estimate chlorophyll, flavonoid (flavonols and anthocyanins) contents, and nitrogen balance index (NBI), chlorophyll *a* fluorescence was measured using a portable fluorometer FluorPen FP 100 (Photon Systems Instruments; Drasov, Czech Republic). The seedlings were adapted to the dark for 30 min for complete oxidation of the photosynthetic electron transport system of the leaves. Then, in the absence of photosynthetically active radiation, the seedlings were quickly removed from the tubes and subjected to a pulse of 3,000 μmol^−2^ s^−1^ of blue light (450 nm), measuring the minimum fluorescence (F_o_) at 50 μs when all the PSII reaction centers were open (defined as step O), followed by step J (at 2 ms), step I (at 30 ms), and the maximum fluorescence (F_m_) when all PSII reaction centers were closed (known as step P), as shown in Supplementary Figure 2. These values were used to calculate various bioenergetic indices of photosystem II according to Strasser et al. (2004), and detailed descriptions of the parameters used in this study are summarized in Table 1.

**Table 1:** Selected JIP parameters from analysis of the OJIP curves used in the analysis of *Brosimum gaudichaudii* seedlings grown *in vitro* under different LED spectra for 50 days.

**Terms and formulas**	**Illustrations**
**Original parameters extracted from the fluorescence transient O-J-I-P**	
F_0_	Initial fluorescence
F_M_	Maximum fluorescence
F_t_	Fluorescence at time t after start of actinic illumination
F_20μ*s*_	Minimum fluorescence signal measured at 20 μs (corresponds to F_0_)
F_300μ*s*_	Fluorescence intensity at 300 μs
F_J_ = F_2ms_	Fluorescence intensity at 2 ms
F_I_ = F_30ms_	Fluorescence intensity at 2 ms
F_P_ = F_300ms_ ≈ F_M_	Fluorescence intensity at 300 ms
Area	Total complementary area between the fluorescence induction curve and F = F_M_
**Calculated parameters**	
F_V_	Variable fluorescence
F_V_/F_0_	Ratio of photochemical to non-photochemical quantum efficiencies (PSII potential activity)
M_0_ = d_V_/dt_0_ = 4 × [(F_300μ*s*_ – F_0_)/(F_M_ – F_0_)]	Net rate of photosystem II closure
V_J_ = (F_2ms_ – F_−0_)/(F_M_ – F_−0_)	Relative variable fluorescence at 2 ms (point J)
V_t_ = (F_t_ – F_−0_)/(F_M_ – F_−0_)	Relative variable fluorescence at time t
**Density of reaction centers**	
RC/CS_M_	Total number of active reaction center
**Quantum yields and probabilities**	
ϕP_−0_ = TR_−0_/ABS = [1 – F_−0_/F_M_] = F_V_/F_M_	Maximum quantum yield of primary photochemistry at (*t* = 0)
ϕE_−0_ = ET_−0_/ABS = [1 – (F_O_/F_M_)]. ψ_−0_ = ϕP_−0_. ψ_1_	Quantum yield of electron transport (at *t* = 0)
ϕD_−0_ = 1 – ϕP_−0_ = (F_O_/F_M_)	Quantum yield of energy dissipation (at *t* = 0)
δR_−0_ = (1 – V_I_)/(1 – V_J_)	Efficiency/probability with which an electron from the intersystem electron carriers moves to reduce end electron acceptors at the PSI acceptor side (RE)
?E_−0_ = ET_−0_/TR_−0_ = (1 – V_J_)	Probability (at *t* = 0) that a trapped exciton moves an electron into the electron transport chain beyond ${\text{Q}}_{\text{A}}^{-}$
**Performance indexes**	
PIABS = $\left(\frac{RC}{ABS}\right).\left(\frac{\varphi Po}{1-\varphi Po}\right)\left(\frac{\psi Eo}{1-\psi Eo}\right)$	Performance index on absorption basis

### *In vitro* Photosynthetic Rate

The photosynthetic rate evaluations were performed according to the method proposed by Costa et al. (2014) for *in vitro* plants, with the culture container itself being the measuring chamber, which allowed the measurement of the entire plant. Seedlings were kept in the dark for 8 h (overnight). Before analysis, they were exposed for 60 min to growth irradiance and immediately evaluated. The measurements were performed in a climate-controlled room using the gas exchange analyzer set from Qubit Systems (Kingston, ON, Canada). The reference air was obtained from a CO_2_/N_2_ cylinder with a standard concentration of 400 μmol mol^−1^. The air flow rate was adjusted to 300 mL min^−1^, the reference air temperature was maintained at 25 ± 1°C, and the relative humidity was adjusted to 60 ± 5%. The measurement irradiance used was 500 μmol m^−2^ s^−1^.

### Morphoanatomical Characterization of Leaves

For the morphoanatomical and micromorphometric analyses, leaf samples of 3 cm^2^ were collected from the central region of the last fully expanded leaf. First the samples were fixed in 70% FAA [formaldehyde:ethanol:acetic acid (4%:50%:5%)] solution (Johansen, 1940) for 24 h. Then the plant material was dehydrated in an ascending ethanol series (70–100%), preinfiltrated, and infiltrated with historesin (Leica Microsystem, Mensheim, Germany). Next, the samples were cut into 5-μm-thick sections using a rotary microtome (Model 1508R, Logen Scientific, China), and the sections were stained with toluidine blue polychromatic staining (0.05%) in phosphate buffer (0.1 M, pH 6.8) (O'Brien et al., 1964). The images were obtained under an Olympus microscope (BX61, Tokyo, Japan) coupled to a DP-72 camera using the bright field option. After the images were obtained, morphoanatomical observations of the adaxial and abaxial epidermis, palisade, spongy parenchyma, and mesophyll were made. Micromorphometric measurements of the captured images were done in ImageJ software (v. 1.47, Java Image Processing and Analysis, Houghton, MI, USA). Measurements were taken for 10 observations per replicate for each structure evaluated.

### Histochemical Tests

Starch, proteins, and phenolic compounds were detected histochemically in the central region of the last fully expanded leaf, after 50 days of cultivation. Starch detection was identified by histochemical of the cuts staining using Lugol solution at 10%, for 5–10 min (Johansen, 1940), for proteins the cuts were stained using xylidine ponceau solution 0.1% for 15–30 min (Vidal, 1970), and for phenolic compounds the cuts were placed in a 10% ferric chloride solution for 15–30 min (Johansen, 1940). Lugol solution was used to detect starch (Jensen, 1962), xylidine ponceau for proteins (Vidal, 1970), and ferric chloride for phenolic compounds (Johansen, 1940). The images were obtained under an Olympus microscope (BX61, Tokyo, Japan), and the percentages of starch, phenolic compounds, and proteins were obtained by the contrast difference using ImageJ software.

### Phytochemical Analysis

The extracts were prepared using 0.2 g of dry leaf matter at 35°C with 4 mL of methanol for HPLC (Neon^®^) in an ultrasound bath for 30 min. The filtration was performed through a membrane Millipore (Advantec HP020AN−20 μm). Then, chromatographic analysis was performed using a Shimadzu HPLC with an SPD-M20A photodiode matrix detector (λ = 254 nm) and an LC18 column (25 cm × 4.6 mm, 5 μm, Supelcosil™) coupled to a 2 cm LC18 precolumn (Supelguard, Supelco). The mobile phase consisted of Milli-Q water (A) and acetonitrile (B) at a flow rate of 0.6 ml/min. The oven was adjusted to 30°C. The sample injection volume was 20 μL, and the detection was 254 nm, following a method adapted from Morais et al. (2018). The furanocumarin concentration present in the extracts was calculated from a four data point calibration curve psoralen and bergapten standard, respectively. The concentration was expressed in micrograms of metabolite per gram of leaf matter (μg/g^−1^ LM). The yield was calculated as the dry weight (g) of the leaves multiplied by the production of the metabolite (μg/g^−1^).

### Statistical Analysis

The data were analyzed for normality with the Shapiro-Wilk test followed by analysis of variance. The data were compared by Tukey's test at 5% probability within Sisvar software (Ferreira, 2019). The principal component analysis (PCA) is a multivariate technique that allows the separation of parameters to obtain a more accurate grouping of the samples and to determine the most discriminant spots. The PCA was performed on the correlation matrix of five light qualities and thirty-three response variables. The PC scores (loadings) generated by the analysis which are also known as eigenvectors were used to identify parameters that best-differentiated the light qualities. The analysis was performed using the R factoextra package based on the ggplot2 package (R Core Team, 2018), considering the first two scores of PC, PC1, and PC2, which accounted for the maximum variability of the tested parameters.

## Results

### Growth Parameters and Morphoanatomical Characterization of Leaves

The influence of light on the growth of *Brosimum gaudichaudii* was evaluated and modulation of growth parameters was observed for all five LED spectra (Table 2). The stem length of the seedlings was longer under red light and shorter in seedlings grown under B/R 1:1 or blue light. Seedlings grown under white light had more leaves. The stem dry matter was higher in seedlings grown under white or B/R 3:1 light. The leaf dry matter and total dry matter were higher in seedlings grown under B/R 3:1 light and lower under blue or B/R 1:1 light. The leaf area was higher in seedlings subjected to red light and lower under blue or B/R 3:1 light. The specific leaf area was also relatively higher in seedlings subjected to red light qualities but lower only under B/R 3:1 light (Table 2).

**Table 2:** Growth parameters of *Brosimum gaudichaudii* seedlings grown *in vitro* under different LED spectra for 50 days.

**Characteristics morphological**	**Light quality**				
	**White**	**Blue**	**Red**	**B/R 1:1**	**B/R 3:1**
Stem length (cm)	3.7 ± 0.24^ab^	3.3 ± 0.37^b^	4.6 ± 0.10^a^	2.8 ± 0.31^b^	3.8 ± 0.12^ab^
Leaf number	3.0 ± 0.00^a^	2.0 ± 0, 00^b^	2.0 ± 0.00^b^	2.4 ± 0.24^ab^	2.7 ± 0.37^ab^
Leaf dry matter (mg)	20.7 ± 1.79^ab^	17.9 ± 2.03^b^	22.6 ± 0.76^ab^	20.3 ± 1.61^ab^	28.8 ± 2.04^a^
Stem dry matter (mg)	34.2 ± 1.03^a^	25.0 ± 2.03^b^	25.2 ± 1.28^b^	20.7 ± 1.16^b^	33.1 ± 2.03^a^
Total dry matter (mg)	55.0 ± 2.10^ab^	43, 0 ± 3.64^c^	47.8 ± 1.68^bc^	41.1 ± 1.79^c^	62.0 ± 3.46^a^
Total leaf area (cm^2^)	4.4 ± 0.34^ab^	3.9 ± 0.12^b^	5.2 ± 0.03^a^	4.0 ± 0.08^ab^	3.8 ± 0.48^b^
Specific leaf area (cm^2^ g^−1^)	0.07 ± 0.00^bc^	0.09 ± 0.01^ab^	0.11 ± 0.00^a^	0.10 ± 0.01^ab^	0.06 ± 0.01^c^

*Mean ± SE (n = 5). Values with the same letters do not differ statistically by the Tukey test at 5%. ± Indicates the standard error of the treatments*.

**Figure 2:**
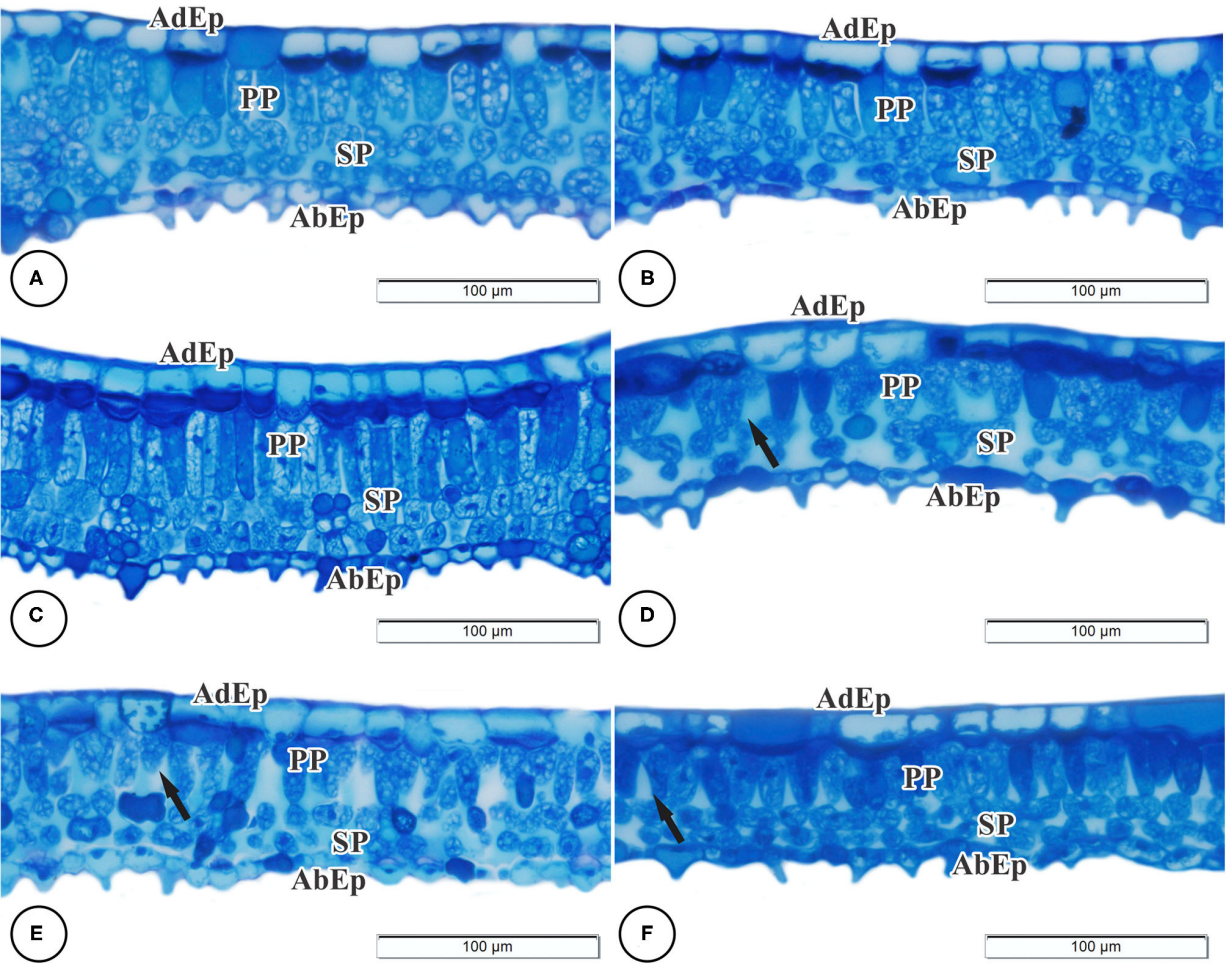
Light microscopy of cross-sections of the leaves of *Brosimum gaudichaudii*. Treatments: white **(A,B)**, blue **(C)**, red **(D)**, B/R 1:1 **(E)**, and B/R 3:1 **(F)**. AdEp, adaxial epidermis. AbEp, abaxial epidermis. PP, palisade parenchyma. SP, spongy parenchyma. Black arrows indicate changes in cell structure. Scale bar = 100 μm.

Regarding anatomy, it was possible to observe that the epidermal tissue of *B. gaudichaudii* is composed of cells of various shapes with thick cell walls, straight to slightly curved, and larger on the adaxial surface than the abaxial surface (Figure 2). On the abaxial surface, the shape of the epidermal cells is undifferentiated due to depressions and a high density of trichomes. The dorsiventral mesophyll consists of one layer of palisade parenchyma and two reduced layers of spongy parenchyma. The effects of different light spectra on the anatomical structures of *B. gaudichaudii* leaves were observed in terms of the morphology and organization of the mesophilic cells (Table 3 and Figures 2C,F). The leaves subjected to the red, B/R 1:1, or B/R 3:1 treatment showed larger intracellular spaces in the palisade and spongy parenchyma (Figures 2D–F) than the white light group (Figures 2A,B). Leaves from seedlings grown under red light showed greater investment in leaf tissue, with a greater thickness of the adaxial epidermis than seedlings grown under blue light. The spongy parenchyma size, palisade parenchyma size, mesophyll size, and leaf thickness of seedlings grown under blue light were higher than those in the other groups. The thickness of the abaxial epidermis was not affected by the use of different spectra of LEDs (Table 3).

**Table 3:** Leaf anatomical structure parameters of *Brosimum gaudichaudii* seedlings grown *in vitro* under different LED spectra for 50 days.

**Characteristics morphological (μm)**	**Light quality**				
	**White**	**Blue**	**Red**	**B/R 1:1**	**B/R 3:1**
Adaxial epidermis	19.10 ± 0.57^bc^	18.82 ± 0.58^c^	22.19 ± 0.17^a^	19.82 ± 0.29^bc^	20.59 ± 0.21^ab^
Abaxial epidermis	10.16 ± 0.84^ns^	9.07 ± 0.27^ns^	11.24 ± 0.46^ns^	11.52 ± 0.29^ns^	10.32 ± 0.52^ns^
Spongy parenchyma	26.74 ± 2.29^ab^	29.93 ± 0.26^a^	21.01 ± 0.89^c^	24.68 ± 1.07^abc^	23.0 ± 1.02^bc^
Palisade parenchyma	25.80 ± 0.73^b^	36.13 ± 0.41^a^	22.05 ± 0.81^c^	23.00 ± 0.57^c^	23.71 ± 0.20^bc^
Mesophyll	51.27 ± 2.30^b^	64.95 ± 1.37^a^	43.75 ± 3.16^b^	50.23 ± 2.67^b^	46.77 ± 1.50^b^
Leaf thickness	79.57 ± 3.41^b^	93.14 ± 0.22^a^	77.52 ± 3.62^b^	74.52 ± 3.67^b^	76.40 ± 1.97^b^

*All data were expressed in μm. Mean ± SE (n = 5). Values with the same letters do not differ statistically by Tukey's test at 5%. ± indicates the standard error, and ^ns^ indicates no significance between treatments*.

### Estimation of Chlorophyll, Flavonol, and Anthocyanin Content and Nitrogen Balance Index

In order to assess whether variations in growth parameters were a consequence of changes in the photosynthetic rate, the authors estimated the pigment content, chlorophyll a fluorescence, and also carbon fixation. However, the contents of chlorophyll, flavonoid, and anthocyanin and nitrogen balance index were not affected by the different LED spectra in *B. gaudichaudii* seedlings grown *in vitro* (Table 4).

**Table 4:** Chlorophyll, flavonoid, and anthocyanin content and nitrogen balance index (NBI) *of Brosimum gaudichaudii* seedlings grown *in vitro* under different LED spectra for 50 days.

**Leaf pigments**	**Light quality**				
	**White**	**Blue**	**Red**	**B/R 1:1**	**B/R 3:1**
Chlorophyll	35.9 ± 3.49^ns^	38.9 ± 5.89^ns^	30.1 ± 2.37^ns^	35.1 ± 2.09^ns^	39.1 ± 3.16^ns^
Flavonoids	0.53 ± 0.08^ns^	0.46 ± 0.14^ns^	0.45 ± 0.08^ns^	0.47 ± 0.09^ns^	0.42 ± 0.04^ns^
Anthocyanins	0.47 ± 0.01^ns^	0.52 ± 0.09^ns^	0.49 ± 0.03^ns^	0.49 ± 0.02^ns^	0.48 ± 0.03^ns^
Nitrogen balance index (NBI)	68.3 ± 11.5^ns^	107.5 ± 21.2^ns^	86.0 ± 17.0^ns^	85.9 ± 14.5^ns^	94.7 ± 25.2^ns^

*Mean ± SE (n = 5). ± indicates the standard error, and ^ns^ indicates no significance between treatments*.

### Polyphasic Fluorescence Transients Chl *a* (OJIP)

**Figure 3:**
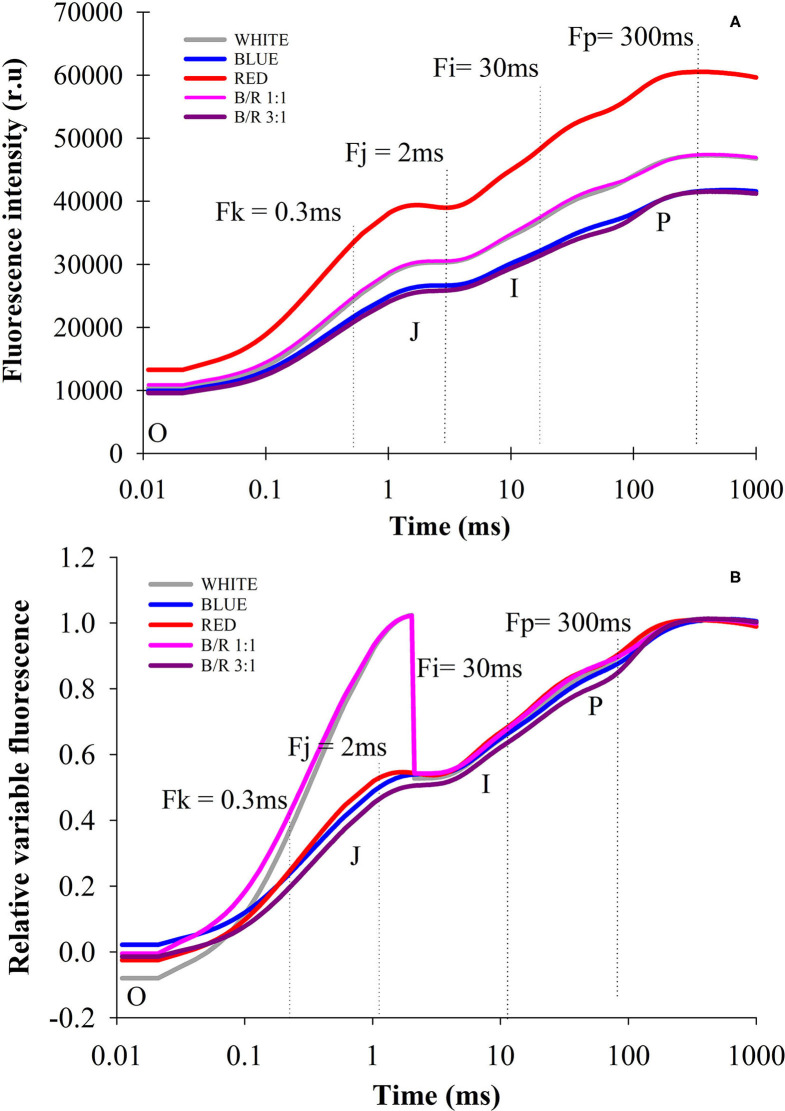
Transients of chlorophyll a fluorescence (OJIP curve), fluorescence intensity **(A)** and relative variable fluorescence **(B)** of *Brosimum gaudichaudii* seedlings at 50 days of *in vitro* cultivation as a function light spectra of white, blue, red, B/R 1: 1 and B/R 3:1.

In the present study, PSII activity was studied by calculating different chlorophyll fluorescence parameters in leaves in the dark-adapted state, showing that all treatments produced typical polyphasic curves with the basic stages of OJIP, from a baseline level (F_o_) to a maximum level (F_m_), and steps J and I well defined (Figure 3A). The initial fluorescence (F_o_) varied more pronouncedly in plants grown under red LEDs compared to white, blue, B:R 1:1 and B/R 3:1 LEDs, showing influence on the initial fluorescence level (F_o_) for the intermediate stages (J and I) and then reached the maximum level (F_m_) (Figure 3A). Changes were observed in the primary electron acceptor quinone Q_A_ of PSII (O-J phase), indicated by changes in the variable relative fluorescence between steps O and P, with a rapid and marked increase in step J for the white and B/R 1:1 treatments, following an immediate decline (Figure 3B), while the extinction of fluorescence controlled by the PSII donor site and the characteristic activity of the water photolysis system (J-Iphase) were not slightly altered, showing no peaks I and P more weak.

**Figure 4:**
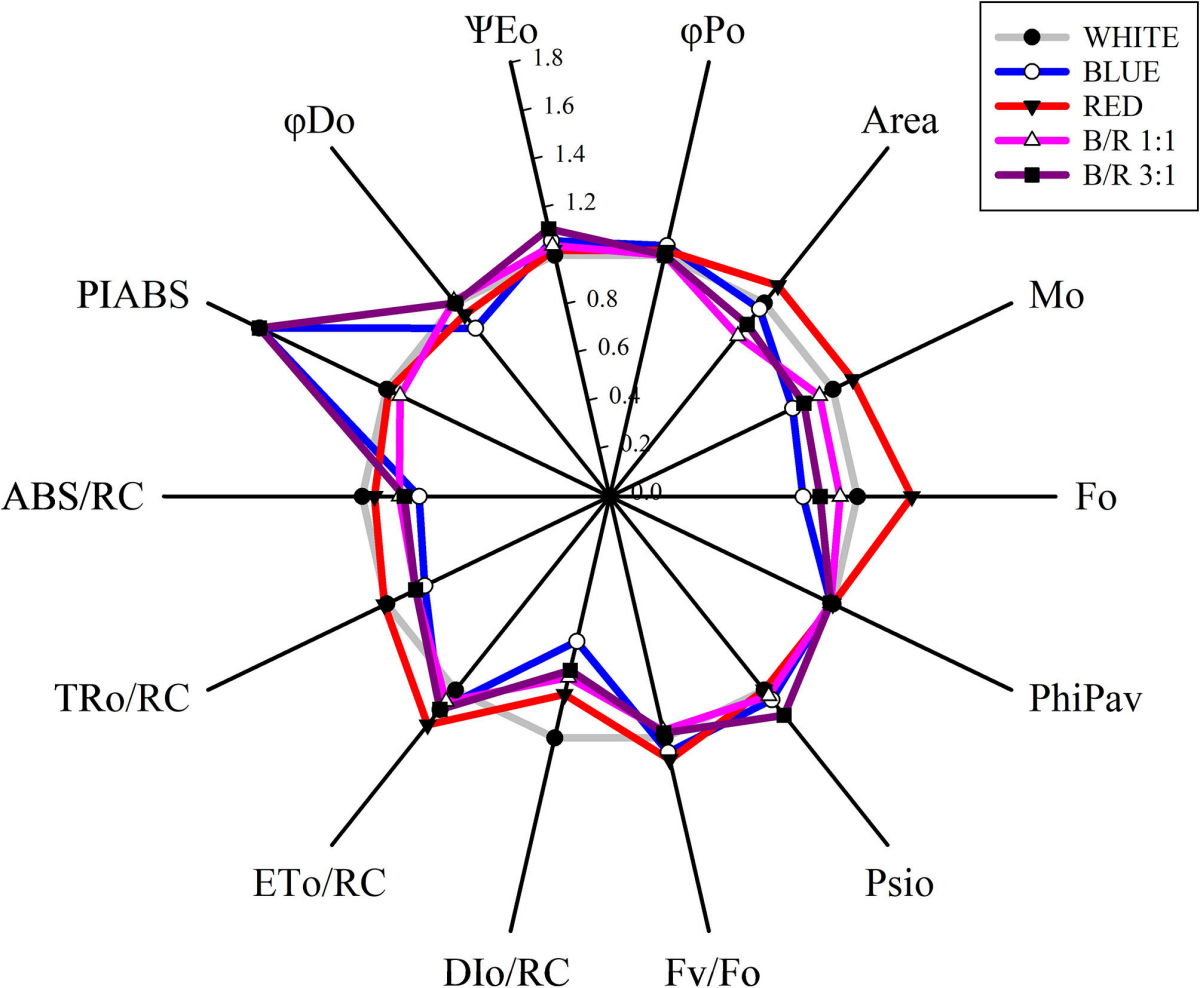
Radar graph of JIP-test parameters as a function of white, blue, red, B/R 1:1 and B/R 3:1light spectra *in Brosimum gaudichaudii* seedlings at 50 days of *in vitro* cultivation. The average value of all parameters of the white treatment was considered as control (each parameter is equal to 1, denoted by the gray circle), and the other parameters in the other treatments were expressed by the fraction of the average value in the white treatment. Individual data points are the mean value of five seedlings.

Through the analysis of the JIP-test, it was found that most parameters based on the fluorescence emission kinetics did not vary according to the LED spectra (Table 5). The parameters of the JIP-test were presented as a function of the spectra, in which case the data were normalized in relation to the control (white) and exposed in Figure 4. Regarding the parameters initial fluorescence (F_o_) and net closing rate of PSII (M_o_) the averages were higher in seedlings under red LEDs, while the area was not significant (Figure 4). Among the different parameters, the specific energy flow for absorption per reaction center (ABS/RC) and the maximum rate of capture of excitons of the PSII per reaction center (TR_O_/RC), increased and were higher in plants grown under white and red LEDs. However, the increases in ABS/RC and TR_O_/RC were accompanied by an increase in the specific dissipated energy flux at the level of the antenna chlorophylls (DI_O_/RC), which resulted in a reduction in the photosynthetic performance index (PI_ABS_) that was promoted in seedlings grown under blue light and B/R 3:1 (Table 5 and Figure 4).

**Table 5:** Initial fluorescence (F_O_); area between OJIP curve and the line F = F_M_ (Area); maximum fluorescence (F_m_); effective quantum yield of photochemical energy conversion (F_v_/F_o_); maximum quantum yield of primary photochemistry (ϕ_Po_); quantum yield, i.e., the probability, at *t* = 0, that a trapped exciton moves an electron into the electron transport chain beyond Qa_−_ (PS_Io_); quantum yield of electron transport (ψ_Eo_); quantum yield of energy dissipation in the form of heat (ϕ_Do_); mean quantum yield of primary photochemistry (ϕ_Pav_); photosynthetic performance index (PI_ABS_); specific absorption flux per reaction center (ABS/RC); maximum exciton capture rate of PSII (T_Ro_/RC); electron transport flux (in addition to Qa) per reaction center at *t* = 0 (E_To_/RC); and specific dissipated energy flux at the level of the antenna chlorophylls (D_Io_/RC) in *Brosimum gaudichaudii* seedlings grown *in vitro* under different LED spectra for 50 days.

**Parameters of chlorophyll fluorescence**	**Light quality**				
	**White**	**Blue**	**Red**	**B/R 1:1**	**B/R 3:1**
F_O_	11814.60 ± 1627.90^ab^	9257.50 ± 459.32^b^	14428.60 ± 1151.91^a^	10989.00 ± 288.45^ab^	10039.60 ± 182.44^b^
Area	18693050.50 ± 237824.4^ns^	18181991.60 ± 2489021.66^ns^	20408156.25 ± 755826.77^ns^	15458199.75 ± 370744.18^ns^	16660992.20 ± 1825688.20^ns^
M_O_	1.09 ± 0.08^ab^	0.89 ± 0.04^b^	1.19 ± 0.07^a^	1.03 ± 0.04^ab^	0.94 ± 0.04^b^
F_v/_F_o_	2.98 ± 0.26^ns^	3.15 ± 0.09^ns^	3.24 ± 0.24^ns^	2.89 ± 0.06^ns^	2.93 ± 0.04^ns^
ϕ_Po_	0.74 ± 0.02^ns^	0.78 ± 0.00^ns^	0.76 ± 0.01^ns^	0.74 ± 0.00^ns^	0.75 ± 0.00^ns^
PS_Io_	0.46 ± 0.01^ns^	0.49 ± 0.03^ns^	0.46 ± 0.01^ns^	0.48 ± 0.03^ns^	0.52 ± 0.01^ns^
ψ_Eo_	0.35 ± 0.01^ns^	0.37 ± 0.03^ns^	0.35 ± 0.02^ns^	0.36 ± 0.02^ns^	0.38 ± 0.02^ns^
ϕ_Do_	0.26 ± 0.02^ns^	0.22 ± 0.00^ns^	0.24 ± 0.01^ns^	0.26 ± 0.00^ns^	0.26 ± 0.00^ns^
ϕ_Pav_	0.95 ± 0.00^ns^	0.94 ± 0.00^ns^	0.95 ± 0.00^ns^	0.95 ± 0.00^ns^	0.94 ± 0.00^ns^
PI_ABS_	1.01 ± 0.21^ab^	1.59 ± 0.13^a^	1.00 ± 0.15^ab^	0.95 ± 0.05^b^	1.59 ± 0.12^a^
ABS/RC	3.07 ± 0.24^a^	2.36 ± 0.05^b^	2.93 ± 0.16^ab^	2.62 ± 0.11^ab^	2.54 ± 0.12^ab^
T_Ro_/RC	2.20 ± 0.13^a^	1.84 ± 0.04^b^	2.22 ± 0.08^a^	1.91 ± 0.01^ab^	1.92 ± 0.09^ab^
E_To_/RC	0.89 ± 0.05^ns^	0.97 ± 0.02^ns^	1.05 ± 0.01^ns^	0.94 ± 0.08^ns^	0.97 ± 0.07^ns^
D_Io_/RC	0.87 ± 0.05^a^	0.52 ± 0.01^b^	0.71 ± 0.09^ab^	0.65 ± 0.05^ab^	0.62 ± 0.04^ab^

*Mean ± SE (n = 5). Values with the same letters do not differ statistically by Tukey's test at 5%. ± indicates the standard error, and ^ns^ indicates no significance between treatments*.

### *In vitro* Photosynthetic Rate

**Figure 5:**
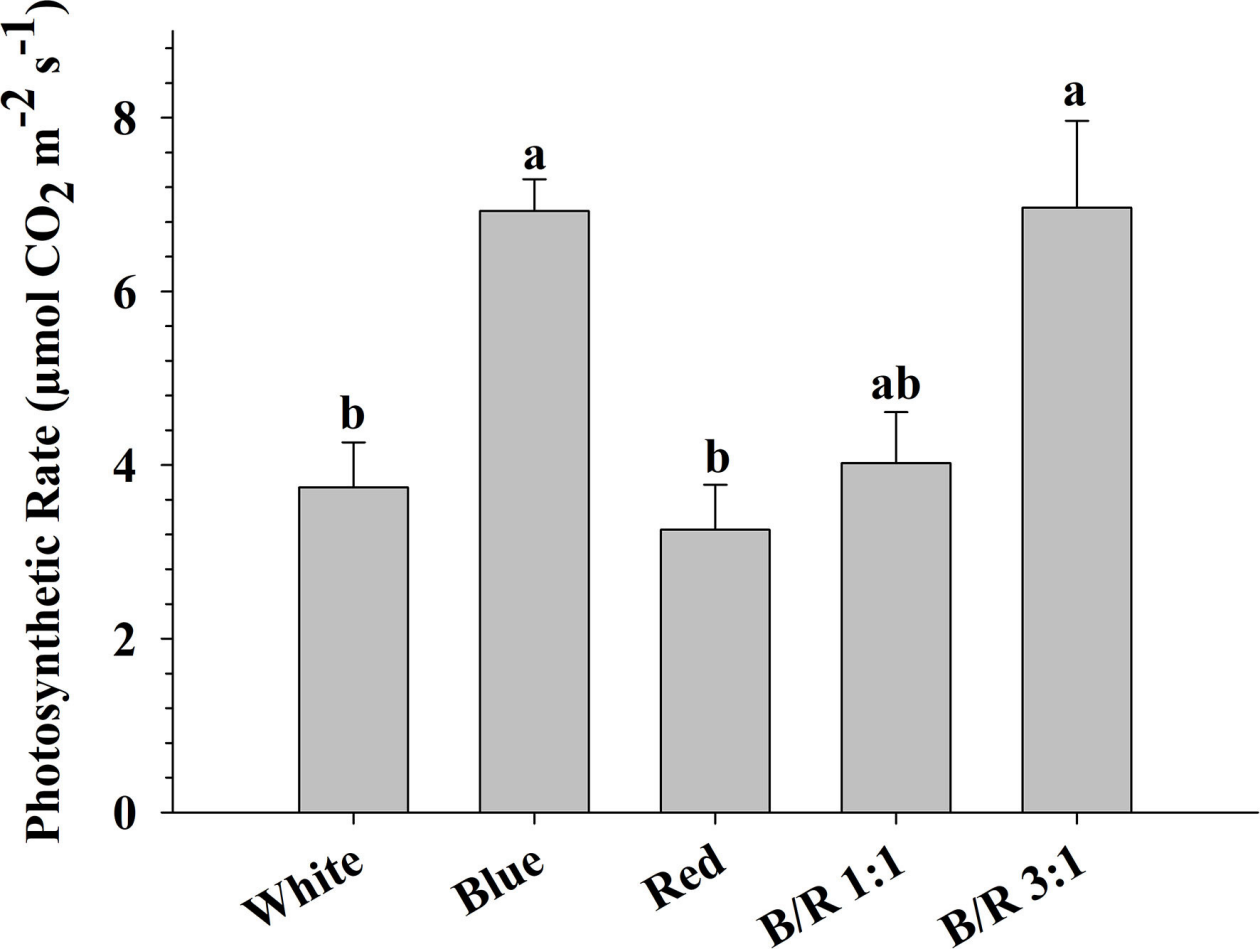
Photosynthetic rate (μmol CO_2_ m^−2^ s^−1^) of *Brosimum gaudichaudii* seedlings grown *in vitro* under different LED spectra for 50 days. Means followed by the same letter do not differ according to Tukey's test at 5% probability. The values are presented as the mean ± standard error (*n* = 5).

The maximum photosynthetic rates (respectively, 6.96 and 6.93 μmol CO_2_ m^−2^ s^−1^) were observed in *B. gaudichaudii* seedlings grown *in vitro* under B/R 3:1 or blue light, while the seedlings grown under light white or red showed the lowest photosynthetic rates (respectively, 3.74 and 3.26 μmol CO_2_ m^−2^ s^−1^) (Figure 5). It is interesting to note that although the plants of the treatments with white light or B/R 3:1 had similar photosynthetic rates and photosynthetic performance index, the accumulation of total dry matter (Table 2) was superior only in the B/R 3: 1 treatment. In view of these results and aiming to evaluate possible carbon deviations between different metabolic routes, the authors performed the detection of starch, proteins, phenolic compounds, and furanocoumarins.

### Histochemical Tests

**Figure 6:**
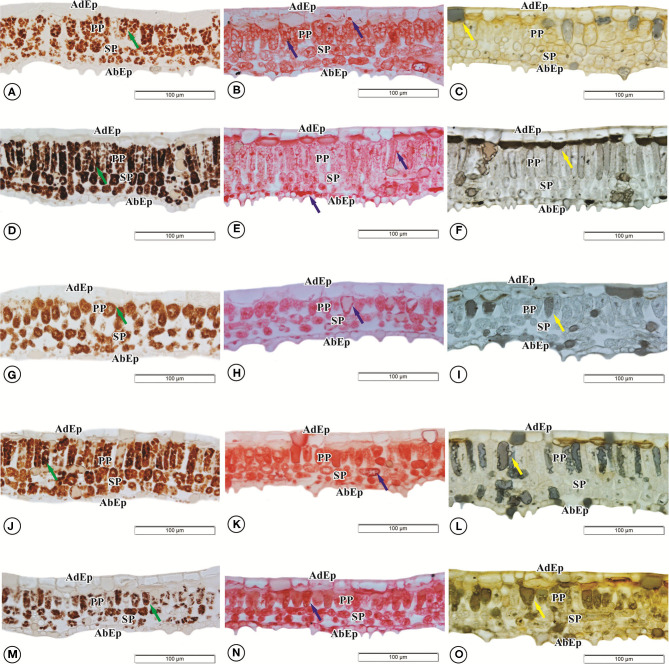
Accumulation of starch **(A,D,G,J,M)**, protein **(B,E,H,K,N)**, and phenolic compounds **(C,F,I,L,O)** in *Brosimum gaudichaudii* leaves cultivated *in vitro* under different spectra of LEDs for 50 days: **(A–C)** white, **(D–F)** blue, **(G–I)** red, **(J–L)** B/R 1:1, and **(M–O)** B/R 3:1. The green arrows indicate the presence of starch. Blue arrows indicate the presence of proteins. Yellow arrows indicate the presence of phenolic compounds. Scale bar = 100 μm.

**Figure 7:**
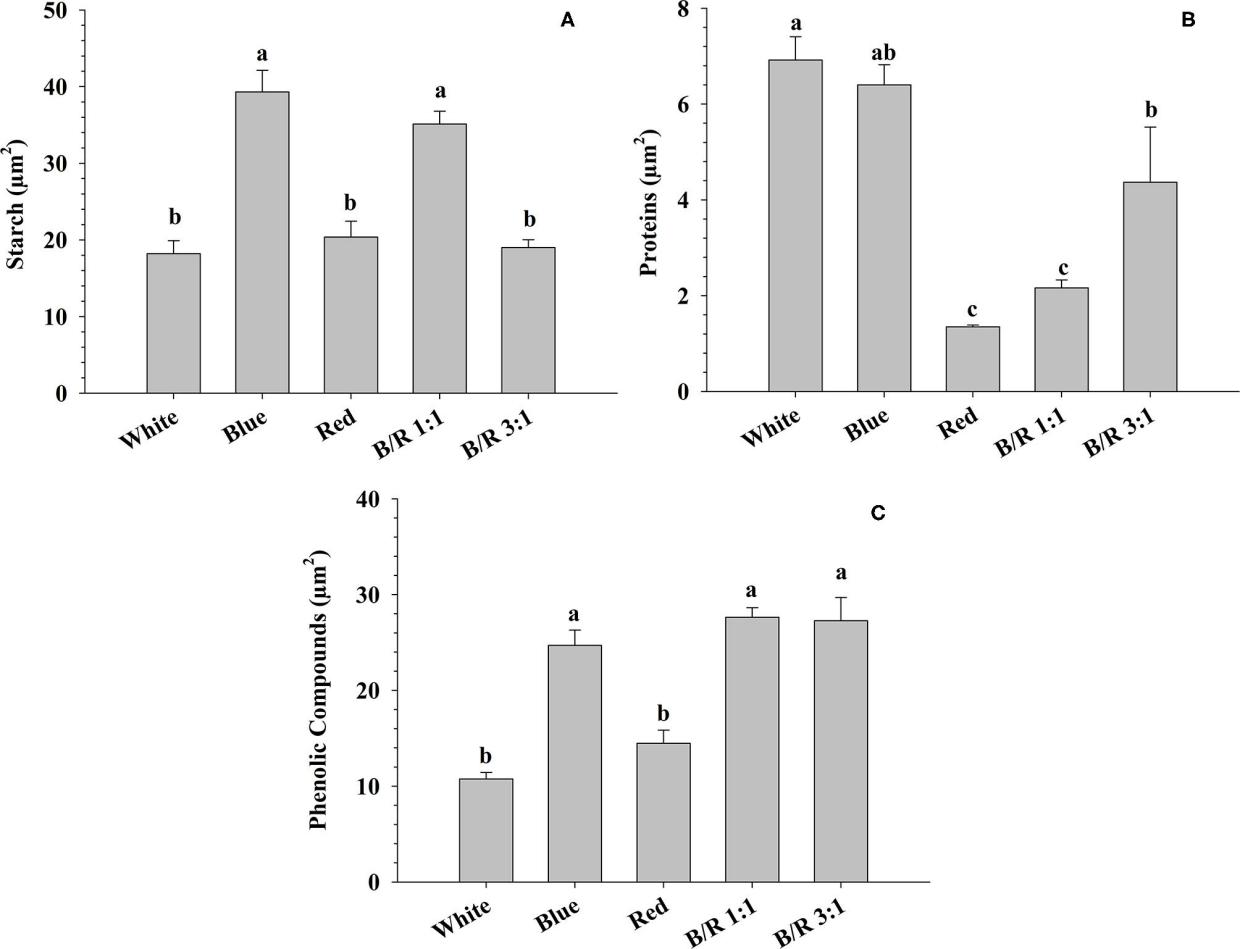
Accumulation of starch, protein, and phenolic compounds in leaf tissues of *Brosimum gaudichaudii* grown *in vitro* under different LED spectra for 50 days. **(A)** Starch (μm^2^), **(B)** protein (μm^2^), and **(C)** phenolic compounds (μm^2^). Means followed by the same letter do not differ according to Tukey's test at 5% probability. The values are presented as the mean ± standard error (*n* = 5).

The results of the histochemical tests performed on *B. gaudichaudii* seedling leaves detected the accumulation of starch in the parenchyma cells under blue or B/R 1:1 light (Figures 6D,J, 7), of protein in the parenchyma and epidermis cells under white treatment (Figures 6B,E, 7B), and of phenolic compounds in the parenchyma and adaxial epidermis cells under B/R1:1, B/R 3:1 and blue light (Figures 6F,L,O, 7C).

### Phytochemical Analysis

**Figure 8:**
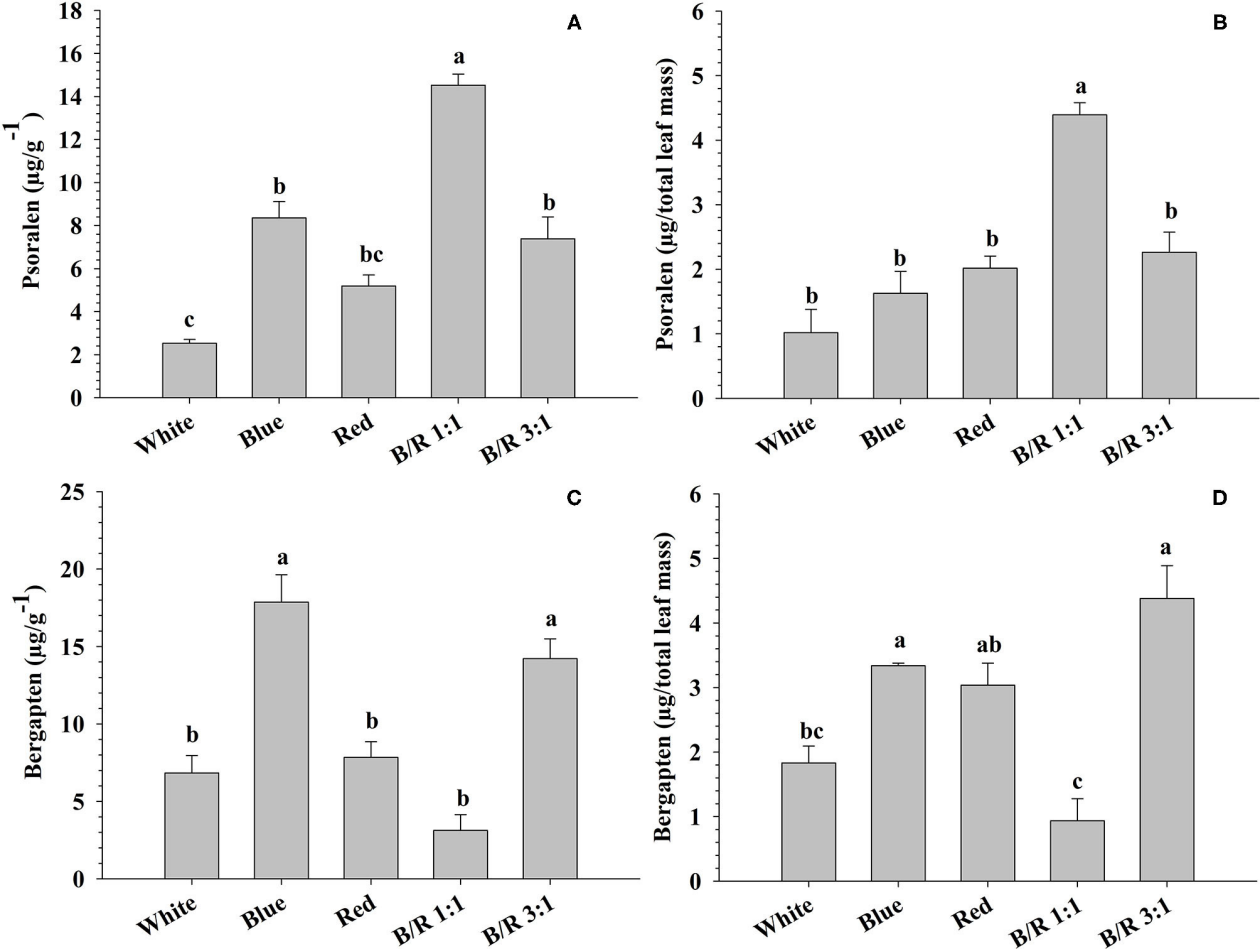
The concentrations (μg/g^−1^) and yields (μg/total leaf mass) of psoralen **(A,B)** and bergapten **(C,D)** in *Brosimum gaudichaudii* leaf tissues grown *in vitro* under different LED spectra for 50 days. Means followed by the same letter do not differ according to Tukey's test at 5% probability. Values are presented as the mean ± standard error (*n* = 3).

As observed for starch, proteins and total phenolic compounds, the variation in light also influenced furanocoumarin concentrations. Indeed, phytochemical analysis of leaves grown under different LED spectra showed significant variations in the concentrations and yields of the secondary metabolites psoralen and bergapten (Figure 8). The highest concentration of psoralen (14.52 μg/g^−1^) was observed in leaves grown under B/R 1:1 light, while the lowest accumulation of psoralen (2.53 μg/g^−1^) occurred in leaves grown under white light (Figure 8A). The highest yield of psoralen (4.39 μg/total leaf mass) was observed under B/R 1:1 light, and the minimum accumulation (1.02 μg/total leaf mass) was observed under white light (Figure 8B). Leaves grown under blue light or B/R 3:1 showed higher accumulation of bergapten (17.86 and 14.21 μg/g^−1^) than other leaves (Figure 8C). Higher yields of bergapten (respectively, 4.38 and 3.34 μg/total leaf mass) were obtained under B/R 3:1 and blue light, while the least accumulation of bergapten (0.94 μg/total leaf mass) was observed in leaves grown under B/R 1:1 light (Figure 8D).

### Principal Component Analysis

**Figure 9:**
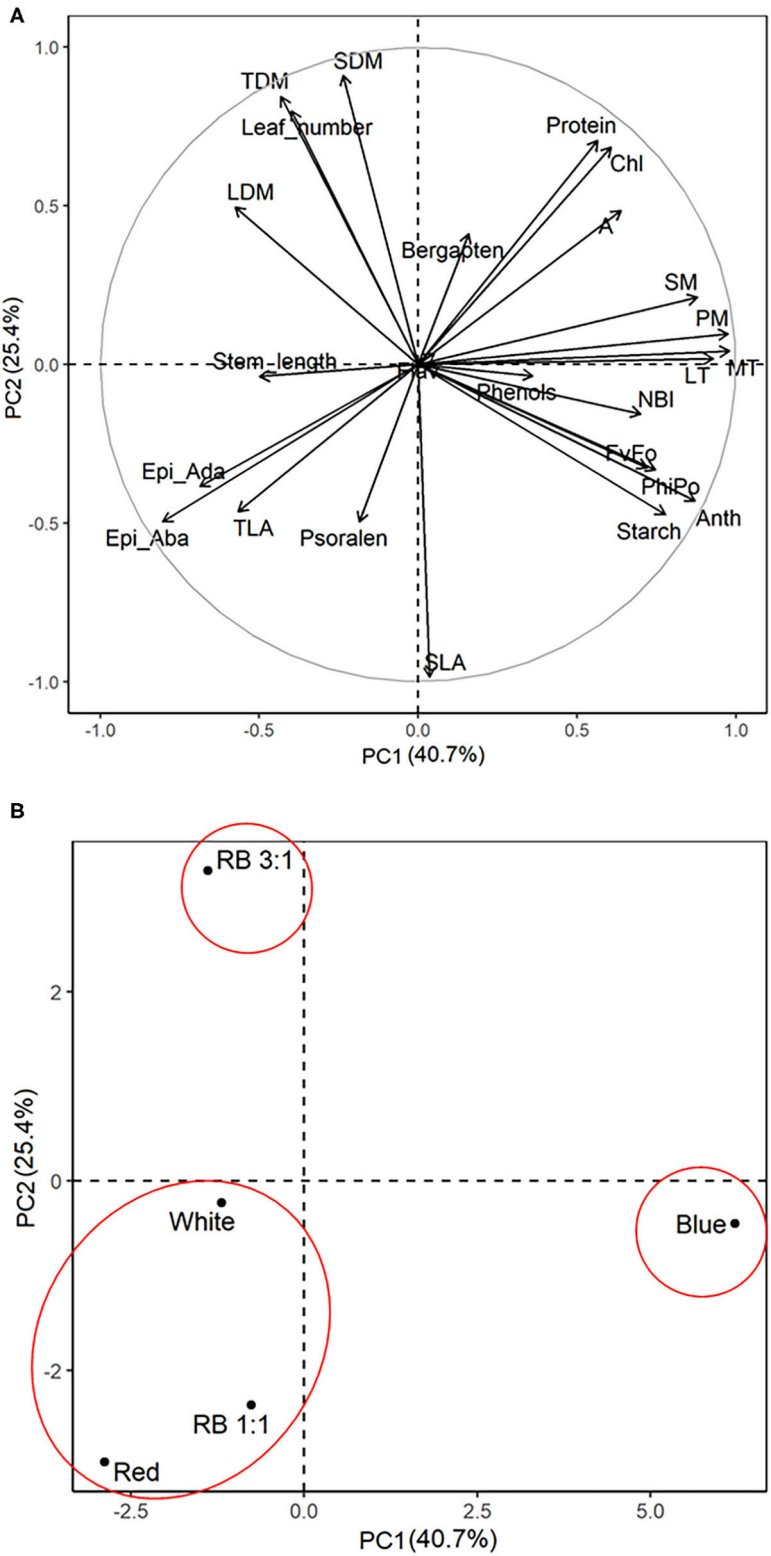
Principal component analysis of biometric, physiological, anatomical, and metabolic characteristics associated with different light qualities. The correlation coefficients for all the analysis **(A)** and the segregation of the experimental groups **(B)** were plotted in the first two component spaces. Leaves, number of leaves; length, stem length; LDM, leaf dry matter; SDM, stem dry matter; TLA, total leaf area; TDM, total dry matter; SLA, specific leaf area; Chl, chlorophyll; Flav, flavonoids; Anth, anthocyanin; NBI, nitrogen balance index; FvFo, effective quantum yield of photochemical energy conversion; ϕPo, maximum quantum yield of primary photochemistry; A, photosynthetic rate; EpiAda, adaxial epidermis; EpiAba, abaxial epidermis.

Loading plots of principal components 1 and 2 of the PCA results obtained from *B. gaudichaudii* grown under different light conditions are shown in Figures 9A,B. Based on the PCA, three groups of seedlings were identified. Individuals of *B. gaudichaudii* grown in white, red, and B/R 1:1 light were grouped together, showing that these plants had similar morphoanatomical, physiological, and biochemical characteristics. In the opposite direction to this group were located the seedlings grown under blue light, indicating that this wavelength, when provided alone, resulted in contrasting responses to those observed under white light. The *B. gaudichaudii* individuals cultured in B/R 3:1 light, in turn, showed different characteristics than the two other groups of plants.

## Discussion

Light is one of the main modulators of plant growth and development, influencing various aspects of plant metabolic processes. In addition to activating and regulating primary metabolism, the quality of light is also particularly important in the intensification of secondary metabolism pathways. Indeed, monochromatic light, as well as combinations of wavelengths, can have significant effects on the production of different secondary metabolites (Landi et al., 2020). Nevertheless, few studies have targeted the improvement of *in vitro* culture techniques for plants from the Brazilian Cerrado to increase the production of bioactive compounds of medicinal interest. In the present study, light affected the morphological and physiological characteristics of *Brosimum gaudichaudii*, including increasing the production of furanocoumarins in the leaves of the seedlings, which may contribute to a more sustainable exploitation of this species.

### Different Light Spectra Influence the Growth and Morphoanatomy of *Brosimum gaudichaudii*

The main changes observed in growth parameters in *B. gaudichaudii* were triggered by red light. This wavelength caused, for example, stem elongation (Table 2 and Figure 9), a result that contrasts with what is generally observed in the literature. In fact, stem elongation is usually associated with blue light (Kong et al., 2018, 2019), while red light has been identified as responsible for inducing decreases in the concentration of indo-3-acetic acid (IAA; Liu et al., 2014). Even so, red light can also induce stem elongation (Claypool and Lieth, 2020; Manivannan et al., 2021), and it is important to consider that the sensitivity to different wavelengths varies from species to species and with the experimental conditions (Liu et al., 2014), although the mechanisms involved in these differential responses are not yet fully understood (Demotes-Mainard et al., 2016). Regarding leaves, both the total leaf area and the specific leaf area were higher in seedlings grown under red light. Similar results were observed in lettuce (Borowski et al., 2015) and *Lepidium sativum* (Ajdanian et al., 2019), and this greater investment in leaf area has been indicated as a response to the lower photosynthetic rate exhibited by seedlings grown under red light (Puglielli et al., 2017).

The highest values for specific leaf area in *B. gaudichaudii* when cultured under red light are closely correlated with the anatomical changes observed under this treatment, since red light, compared to blue light, reduced the spongy parenchyma, palisade parenchyma, mesophyll, and leaf thicknesses (Table 3), resulting in the production of larger and thinner leaves. These results indicate the stimulation of the phytochrome to promote greater capture of light energy to boost the photosynthesis of seedlings in response to the low-red/far-red ratio (Miao et al., 2019; Zou et al., 2019), as observed in seedlings of *Solanum lycopersicum* L., *Scrophularia takesimensis, Solanum tuberosum*, and *Myrtus communis* L. Nakai (Jeong and Sivanesan, 2015; Cioć et al., 2018; Naznin et al., 2019; Chen et al., 2020). Contrary to what was observed for red light, the blue light individually increased the thickness of the palisade parenchyma, of the mesophyll and, consequently, of the leaf. Changes in leaf thickness due to increases in palisade parenchyma thickness have been correlated with increases in photosynthesis, since the structure of this tissue favors the photosynthetic process (Zheng and Van Labeke, 2017).

In our study, the 3:1 of blue to red light increased the biomass production (Table 2), which was probably due to a higher photochemical efficiency and higher photosynthetic capacity, as observed for other species, such as *Hordeum vulgare, Zea mays*, and *Oryza sativa* L. (Bukhov et al., 1995; Matsuda et al., 2004). The B/R 3:1 ratio seems to be a more suitable culture environment than B/R 1:1, blue, red, or white light, since seedlings grown under favorable growth conditions have higher dry matter accumulation in the shoots, as previously observed for other plant species (Thwe et al., 2020). In plants exposed to monochromatic blue light, on the other hand, the increase in photosynthetic rate was not reflected in higher biomass accumulation (Table 2 and Figure 5). This reduction in biomass may be related to the greater accumulation of storage compounds, such as starch (Figure 7), and the redirecting of carbohydrates to other metabolic pathways, as blue light can promote the diversion of carbon to the respiratory process (Kowallik and Gaffron, 1966; Hogewoning et al., 2010). It is also possible that seedlings grown under monochromatic blue light invest a higher proportion of carbon in defense mechanisms, since this wavelength can induce the generation of reactive species in plant cells, leading to an increase in the concentration and activity of antioxidants (Manivannan et al., 2015).

### Influence of Light on the Physiological Characteristics of *B. gaudichaudii*

The seedlings grown under B/R 3:1 and under monochromatic blue light showed a better photosynthetic performance index (Table 5) and higher photosynthetic rate (Figure 5), indicating that the blue wavelength increases the photosynthetic potential of *B. gaudichaudii*. Consistent with these data, seedlings of *Pfaffia glomerata* (Silva et al., 2020) and *Fagus sylvatica* (Košvancová-Zitová et al., 2009) also showed higher photosynthetic rates when grown *in vitro* under a higher ratio of blue light. The increase in the photosynthetic rate in response to blue light is probably due to the combined effect of this wavelength on different processes associated with carbon fixation, such as the induction of stomatal opening (Inoue and Kinoshita, 2017), the activation of genes encoding chloroplast proteins (Yu et al., 2010), the faster activation of Rubisco (Košvancová-Zitová et al., 2009), and even the movement of chloroplasts, to allow more efficient light capture (DeBlasio et al., 2003).

Despite the involvement of blue light in several aspects of the photosynthetic process, monochromatic blue light can reduce photosynthetic efficiency by promoting the cyclic electron flow to avoid the occurrence of photoinhibition, since this wavelength has a high energy content (Yang et al., 2018). In this study, the moderate light intensity (100 μmol m^−2^ s^−1^) used during seedling growth effectively activated photosynthesis without inducing photoinhibition. In addition, in *B. gaudichaudii*, monochromatic blue light or the combination of blue and red light induced the accumulation of metabolites that help the plant avoid photoinhibition, such as phenolic compounds, which accumulated mainly in the adaxial epidermis and parenchyma (Figures 6, 7; Takahashi and Badger, 2011). Phenolic compounds can act as filters that absorb excess radiant energy, protecting internal tissues from possible damage caused by radiation (Solecka, 1997). Phenolic compounds can also act in the reduction or elimination of free radicals (Zheng and Van Labeke, 2017). These results are consistent with observations made in other species, such as *Agastache rugosa* and *Rehmannia glutinosa* (Manivannan et al., 2015; Zielińska et al., 2019). In turn, the accumulation of protein observed in leaves grown under white light (Figures 6B, 7B) might be associated with reduced damage to PSII, since these seedlings had higher ABS/RC and T_Ro_/RC than the other groups, which resulted in the need for greater protection, such as protein accumulation and adjustment of energy dissipation (D_Io_/RC), reducing the pressure on the D_1_ protein of PSII (Mlinarić et al., 2017).

In this study, the seedlings of *B. gaudichaudii* demonstrated typical OJIP phases for all LED spectra, indicating that the seedlings were photosynthetically active. The results clearly stated that the different spectra influenced the photosynthetic apparatus (Figure 3A). Changes in initial fluorescence (F_O_) observed under red LEDs (Figure 3B), may be related to energy losses in the pigments of the PSII antenna complex (Rosa et al., 2018), which may be indicative of partial inhibition of the PSII reaction center, which in turn may have inhibited the flow of electrons from the primary quinone electron acceptor (Q_A_) to the secondary quinone electron acceptor (Q_B_), decreasing the efficiency of the energy captured in the PSII, indicating damage to the D_1_ protein in the reaction center (Strasser et al., 2004; Bano et al., 2021). This decrease in photosynthetic efficiency may be related to a lower need for trapped energy to close the reaction centers, indicating that there was a reduction in primary photochemical activity (Kumar et al., 2020).

The higher amplitudes of the O-J phases of the variable relative fluorescence for the white and B/R 1:1 treatments, indicated impairment of the photochemical reduction capacity (Figure 3B). Since the O-J band indicates the Q_A_ reduction reaction from pheophytin (Phe), the decrease in electron supply/performance on the acceptor side, decreased the electron transfer efficiency due to the fact that Q_B_ cannot receive the electrons of ${\text{Q}}_{\text{A}}^{-}$ in time, leading to instantaneous accumulation of mass of ${\text{Q}}_{\text{A}}^{-}$ indicating that electron transport beyond ${\text{Q}}_{\text{A}}^{-}$ has been restricted (Gao et al., 2021). Seedlings under blue, red and B/R 3:1 LEDs showed no changes in the amplitude of these bands, indicating that the ability to photochemically reduce Q_A_ was maintained, as they had the necessary kinetic properties to reduce and oxidize the quinone pool (Martins et al., 2015b).

### Production and Accumulation of Furanocoumarins in *B. gaudichaudii* Leaves: What Is the Impact of Light?

Stimulating the production of secondary metabolites by different light conditions *in vitro* is one of the simplest methods to increase the production of bioactive compounds of medicinal and economic importance (Kubica et al., 2020; Manivannan et al., 2021). The effects of different light spectra on the accumulation of secondary metabolites were evaluated in *B. gaudichaudii* with satisfactory results, showing that higher amounts of psoralen were produced in leaves of seedlings grown under the B/R 1:1 light, followed by plants exposed to B/R 3:1 and then to blue light, while the highest concentrations of bergapten were observed under blue or B/R 3:1 light (Figure 8).

The increase in furanocoumarin concentration generally required an increase in the photosynthetic process and/or diversion of carbon into secondary metabolism. Specimens of *B. gaudichaudii* exposed to the B/R 1:1 ratio, for example, accumulated psoralen but showed lower growth and biomass accumulation than plants under white light, which had the lowest furanocoumarin concentration. Indeed, since seedlings of the B/R 1:1 group did not show an increased photosynthetic rate, they needed to divert carbon from other routes to synthesize psoralen, a phenomenon commonly observed in plants that have increased synthesis of secondary metabolites (Caretto et al., 2015). In addition, the production of psoralen was negatively correlated with protein synthesis (Figures 7, 9), reinforcing the hypothesis that secondary metabolism was stimulated at the expense of the primary metabolism and negatively correlated with the concentration of phenolic compounds (Figure 9).

Seedlings exposed to blue or B/R 3:1 light, in addition to presenting high production of psoralen (behind only the specimens exposed to the B/R 1:1 treatment), had the highest concentrations of bergapten, which demanded an increase in the carbon fixation rate. In fact, the bergapten concentration was positively correlated with carbon fixation rate (Figure 9). Although both blue and B/R 3:1 light conditions provided increased photosynthesis, only seedlings grown under B/R 3:1 showed high growth. As discussed above, the activation of other metabolic pathways by monochromatic blue light, such as respiration, together with the increase observed in the synthesis of secondary metabolites, may have contributed to the decrease in growth under this treatment, even with the increase in carbon fixation. In the B/R 3:1 treatment, on the other hand, the higher photosynthetic rate was sufficient to supply carbons for secondary metabolism without compromising primary metabolism.

The results obtained in the present study clearly show that the variation in the light spectrum during *Brosimum gaudichaudii* culturing can induce the synthesis and accumulation of furanocoumarins in leaves, an organ in which these compounds do not naturally accumulate. In fact, culturing seedlings under B/R 3:1 altered their cellular metabolism and increased their photosynthetic rate, which in turn allowed the accumulation of secondary metabolites without compromising plant growth. Plants grown in blue light alone, although they had similar production of secondary metabolites, showed low growth, which indicates the activation of carbon diversion pathways, such as respiration. As far as it was possible to verify, this is the first work that presents a biotechnological methodology for increasing furanocoumarin production in *B. gaudichaudii*. These plants produce large amounts of furanocoumarins but have slow growth and accumulation of psoralen and bergapten in the roots. Thus, in traditional cultivation or in collections in the environment, it is necessary to wait for the slow growth of the plant until sufficient biomass is produced to allow a significant obtaining of the compound of interest; and the obtention of the compounds is carried out by removing the root system, which results in the death of the plant. These factors make traditional cultivation unsuitable to meet the growing market demands for furanocoumarins and make extraction predatory. If the light conditions observed in this experiment were reproduced in *in vitro* cultivation and, later, used in adult plant cultivation, by Outdoor Supplemental Lighting, the leaves of the same plant could supply furanocoumarins several times throughout its life cycle, which is quite significant, since this species lives for many years. In this way, the results presented here provide new prospects for the production of psoralen and bergapten in the leaves of *B. gaudichaudii in vitro*, which will have important implications both for obtaining medicinal compounds and for the conservation of the species.

## Data Availability Statement

The original contributions presented in the study are included in the article/Supplementary Material, further inquiries can be directed to the corresponding author/s.

## Author Contributions

FS was responsible for obtaining the necessary funding. EC, MR, and TO wrote the original draft, revised and edited it, and conducted the experiments. EC, AJ, and ER supervised, validated, and performed the chromatographic analyses. EC, TO, and AR performed the anatomical analyses. EC, FF, and FS helped with managing the project, resources, programs, research supervision, and writing of the article. All authors contributed to the article and approved the submitted version.

## Conflict of Interest

The authors declare that the research was conducted in the absence of any commercial or financial relationships that could be construed as a potential conflict of interest.
