# Supplementary material for: Combinations of Blue and Red LEDs Increase the Morphophysiological Performance and Furanocoumarin Production of Brosimum gaudichaudii Trécul in vitro
Source: Front Plant Sci. 2021 Jul 21;12:680545. doi: 10.3389/fpls.2021.680545 (PMC8334558; doi:10.3389/fpls.2021.680545)
Supplement: Supplementary file 1 [file Data_Sheet.pdf]

Supplementary Material

Combinations of blue and red LEDs increase the morphophysiological performance and furanocoumarin production of *Brosimum gaudichaudii* Trécul *in vitro*

Érica Letícia Gomes Costa[1], Fernanda dos Santos Farnese[1], Thales Caetano de Oliveira[1], Márcio Rosa[1], Arthur Almeida Rodrigues[1], Erika Crispim Resende[2], Ana Helena Januario[3], Fabiano Guimarães Silva[1*]

***** Corresponding Author: fabiano.silva@ifgoiano.edu.br

Figure S1. Light spectra

Figure S2. A simplified schematic diagram of electron transport in photosynthesis modified from (Shevela et al., 2018). The OJ phase is the photochemical process related to the reduction of primary quinone electron acceptors (Q_A) to Q_A^-. A majority of Q_A^- that cannot be timely oxidized by second quinone electron acceptors (Q_B) is accumulated rapidly, causing the fluorescence intensity to increase instantaneously because it only takes ~250 ps for the electron transferring from pheo– to Q_A, but 0.1~0.6 ms from Q_A to Q_B). The intermediate phase JI corresponds to the reduction of plastoquinone (PQ) pool needs much more time, approximately 1~20 ms, due to the physical distance between PSII and cytochrome b6f (Cyt b6f) complex. The IP phase parallels the reduction of acceptors in and around photosystem I (PSI), namely PC+ and P700+.

Figure S1

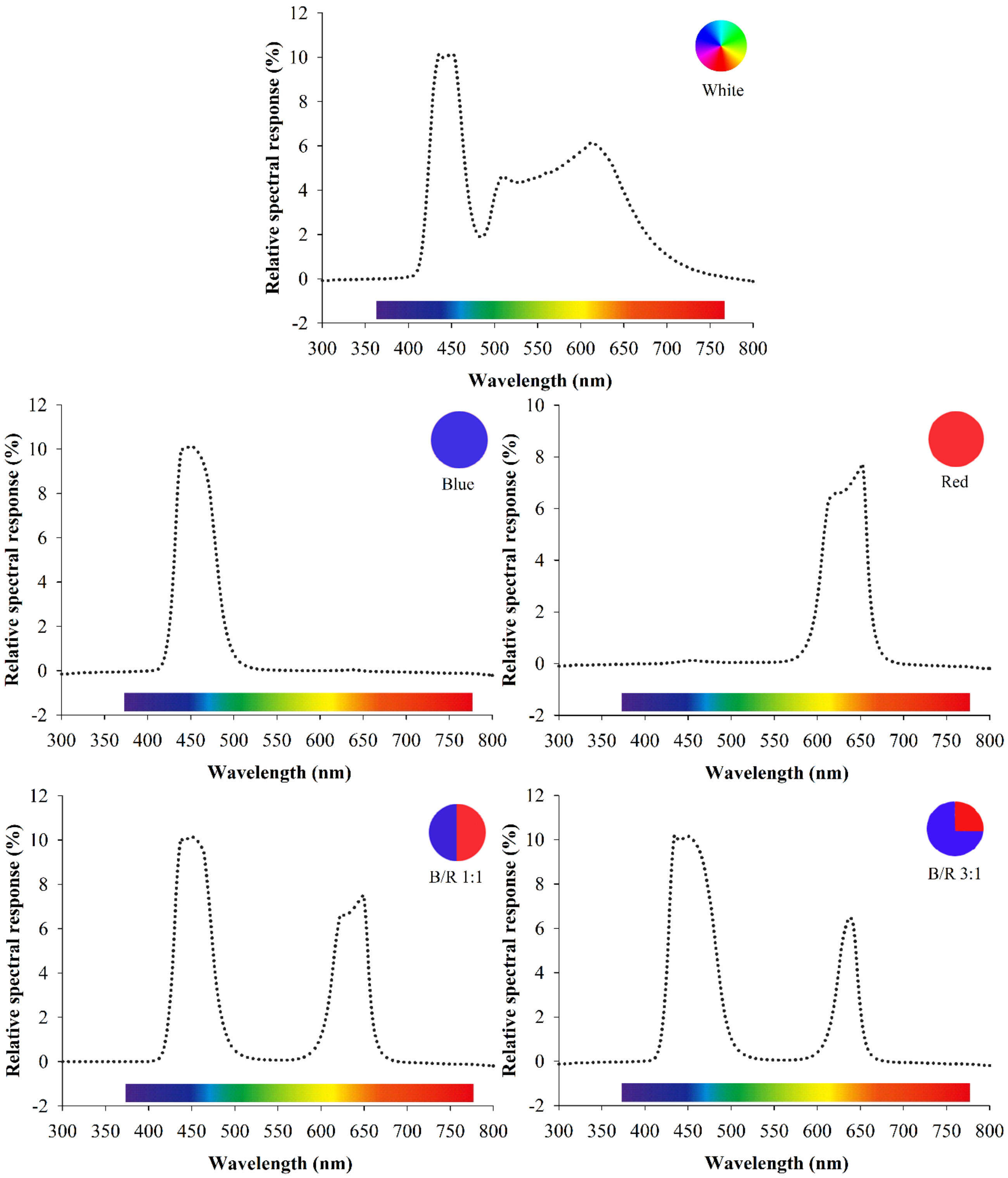
White
Blue
Red
B/R 1:1
B/R 3:1
Relative spectral response (%)
Wavelength (nm)
12
10
8
6
4
2
0
-2
300 350 400 450 500 550 600 650 700 750 800

Figure S2

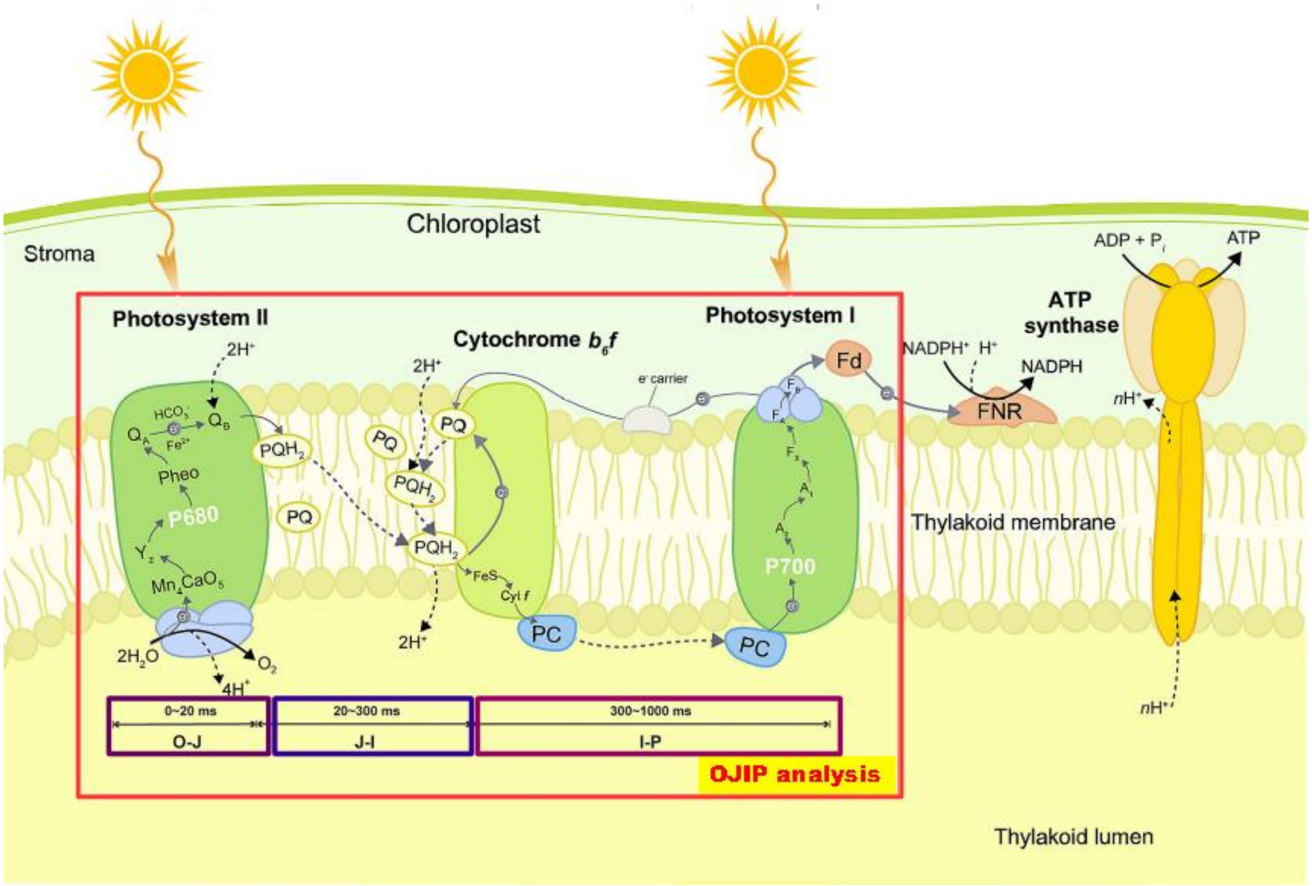
Chloroplast
Stroma
Photosystem II
Cytochrome b_6f
Photosystem I
ATP synthase
ADP + P_i
ATP
$2H^+$
HCO_3^-
Q_A
Q_B
Fe^{2+}
Pheo
P680
Y_Z
Mn_4CaO_5
$2H_2O$
O_2
$4H^+$
PQH_2
PQ
FeS
Cyt f
PC
e⁻ carrier
Fd
F_B
F_A
F_X
A_1
A_0
P700
$NADPH^+$
H^+
NADPH
FNR
nH^+
Thylakoid membrane
Thylakoid lumen
0~20 ms
O-J
20~300 ms
J-I
300~1000 ms
I-P
OJIP analysis
